# Mixed oats and alfalfa improved the antioxidant activity of mutton and the performance of goats by affecting intestinal microbiota

**DOI:** 10.3389/fmicb.2022.1056315

**Published:** 2023-01-09

**Authors:** Yukun Sun, Tingyi Hou, Qingyuan Yu, Chengrui Zhang, Yonggen Zhang, Lijun Xu

**Affiliations:** ^1^College of Animal Science and Technology, Northeast Agricultural University, Harbin, China; ^2^Institute of Agricultural Resources and Regional Planning, Chinese Academy of Agricultural Sciences, Beijing, China

**Keywords:** Albas goat, alfalfa hay, oat hay, fattening performance, slaughter performance, antioxidant capacity

## Abstract

Oat hay and alfalfa hay are important roughage resources in livestock production. However, the effect of the mixture of oat hay and alfalfa hay on the meat quality of Albas goats is unclear. This study aimed to investigate the effects of feeding different proportions of oat hay and alfalfa hay on the growth performance and meat quality of Albas goats. Therefore, 32 goats were fed for 70 days and randomly divided into four treatment groups on the principle of similar weight: whole oat group (OAT), oat alfalfa ratio 3:7 group (OA73), oat alfalfa ratio 7:3 group (OA37) and whole alfalfa group (Alfalfa), with eight goats in each group. Daily feed intake records, feces, feed samples, and rumen fluid collection were made throughout the trial. The goats were weighed on the last day of the trial, and four goats per group were randomly selected for slaughter. Cecum contents, meat samples, and hot carcass weight were collected, and data were recorded. Furthermore, the relationship between the rumen and cecal microbes on performance and meat quality was clarified by analyzing the rumen and hindgut microbiomes. The results showed that feeding alfalfa could significantly reduce the daily weight gain of fattening goats. Compared with the highest group (OA37), the daily weight gain decreased by 19.21%. Although there was no significant change in feed intake in the four treatments, the feed conversion rate of the alfalfa group significantly decreased by 30.24–36.47% compared to the other groups. However, with the increased alfalfa content, MDA decreased significantly, T-AOC was up-regulated, and the antioxidant activity of the fattened goat meat fed with the high alfalfa group was significantly higher than that of the low alfalfa group. Notably, the abundance of *Bacteroidales_unclassified* and *Clostridium* were strongly correlated with T-AOC and MDA. Therefore, increasing the proportion of alfalfa in the diet can affect the antioxidant activity of goat meat by improving the gut microbiota, while an oat-hay mixture can improve the growth performance of livestock.

## 1. Introduction

Between 2020 and 2050, the global average protein demand for red meat, poultry, milk, and eggs will increase by 14%, and the total will increase by 38%, with lamb being one of the essential components of red meat consumption in the world. The most important factors affecting the production efficiency of mutton sheep include the host genetics (breed), gender, growth stage or age, dietary factors, environmental factors, feeding methods, etc. Ruminants such as cattle, sheep, and goats use fiber better than animals with a single stomach. Dietary fiber can stimulate chewing and rumination, promote saliva secretion, enhance rumen fermentation function and improve the quality of animal products. However, too much fiber in the diet will affect the diet’s palatability and reduce the dry matter intake of sheep (Adesogan et al., 2019). Therefore, finding a strategy for choosing high-quality roughage is a crucial way to improve the production efficiency of sheep meat to meet the needs of red meat.

Feeding high-quality roughage is beneficial to maintain rumen homeostasis and reduce the occurrence of metabolic diseases such as rumen acidosis and abomasum displacement (Kenny et al., 2018). At present, high-quality forages such as alfalfa, oat grass, and ryegrass are the primary sources of high-quality protein in animal husbandry production and can provide animals with a variety of vitamins, minerals, and other trace elements. As a high-quality forage with strong stress resistance, alfalfa has many characteristics, such as promoting digestion and absorption of ruminants and maintaining body health, so it is one of the most popular roughages in the world. At the same time, because the content of neutral detergent fiber and lignin in oat grass is lower than that in roughage such as alfalfa, it has good palatability and helps to improve the digestion, absorption, and feed utilization efficiency of ruminants (Radović et al., 2009). It has gradually become an important source of roughage in animal husbandry production in China (Abeysekara, 2003). Combining oats and alfalfa may provide energy and adequate physical fiber for fattening sheep, ensure growth efficiency, and protect rumen health.

Dietary therapy may be compared with concentrate fodder, and the forage-based system usually promotes lean meat, widely raising the accumulation of natural antioxidants. This results in better oxidative stability of pasture-raised beef than concentrate-fed beef (Van Elswyk and McNeill, 2014; McAllister et al., 2020). Several reports have confirmed that grazing beef cattle have the advantage of producing lean meat with more omega-3 fatty acids, which directly affects the balance of antioxidant and pro-oxidative components in muscles (Ponnampalam et al., 2012; Inserra et al., 2014). In addition, the increase in antioxidant properties of meat can effectively inhibit lipid oxidation of shelf-stable meat and prolong its shelf life (Hayes et al., 2011). Meanwhile, supplementing the diet with natural antioxidants can improve the meat quality and its organoleptic properties by depositing them in the muscle with other metabolites of their own, thus performing a probiotic function (Morán et al., 2012). This is essential to improve the meat quality and antioxidant properties of lamb by feeding natural substances or high-quality roughage instead of synthetic agents after slaughter to prolong its shelf life, organoleptic quality, and richness in lipids (Andrés et al., 2014). This is to avoid supplementing with exogenous substances during meat production and processing and to help them meet the market requirements for high-quality meat. Thus, livestock fed roughage rich in dietary forage combinations have the potential to improve livestock meat quality.

The synergistic supply of alfalfa hay, starter culture, and emulsion substitute significantly improved the rumen fermentation capacity of cattle and lambs and changed the rumen microbiota, which was the main reason for the improved growth performance of cattle and lambs (Wu et al., 2021). In addition to rumen fermentation, alfalfa hay can also affect starch and fiber in the rumen bypass, affecting hindgut microbiota and fermentation (Wu et al., 2019). As the main hindgut, the fermentation in the cecum is also an important factor affecting growth performance. The changes in cecal microorganisms lead to the improvement of meat quality. However, the mechanism of how the changes in roughage affect meat quality by altering the gut microbiota is still being determined.

This study hypothesized that different proportions of alfalfa and oat feed would have different performances on fattening goats, and long-term feeding with different mixed proportions of alfalfa and oat would affect rumen fermentation, thereby affecting the changes in cecal microbiota and meat quality of goats. This study aimed to investigate the effects of different alfalfa and oat mixing ratios on fattening goats’ performance and meat quality. It also clarifies the relationship between rumen and cecal microbes on performance and meat quality.

## 2. Materials and methods

The experimental protocol (Protocol number: NEAU- [2011]-9) was approved by the Northeast Agricultural University Animal Care and Use Committee (Harbin, China). The ranch meets the welfare standards of the China Animal Protection and Use Commission.

### 2.1. Experimental design

This experiment was conducted at the Acheng Experimental Base of Northeast Agricultural University from February 2022 to April 2022. Thirty-two healthy and disease-free Arbas male goats aged 6 months (22.63 ± 0.20 kg) were randomly divided into 4 groups with 8 repeats in each group according to the principle of similar body weight. The experimental groups were whole oat (OAT), oat: alfalfa = 7:3 (OA73), oat: alfalfa = 3:7 (OA37), and whole alfalfa (Alfalfa) treatment (All forage were in air-dried samples). All the experimental goats were raised in a single column and drank freely. In this experiment, concentrate and roughage were fed separately at 06:00 and 18:00 daily, and the ratio of concentrate to forage was 65:35. The experiment lasted 70 days, where the prefeeding period was 14 days, and the experimental period was 56 days.

### 2.2. Composition and nutrition level of experimental diet

The diet was composed of concentrate having corn, corn germ meal, sprayed corn husk, DDGS, expanded soybean, and different proportions of alfalfa and oat grass mixed roughage. The dietary composition and nutrition levels are shown in Table 1.

**Table 1:** Composition and nutrient levels of diets (dry matter basis, %).

Nutrient levels	Oat	OA73	OA37	Alfalfa	Concentrate
Dry matter	92.07	92.05	92.03	92.01	90.96
Crude protein (CP)	11.93	11.97	12.02	12.06	16.39
Ether extract (EE)	1.28	1.26	1.23	1.20	3.82
Neutral detergent fiber (NDF)	56.47	56.48	56.50	56.50	30.94
Acid detergent fiber (ADF)	33.19	35.30	38.12	40.23	16.29
Metabolizable energy (MJ/kg)^a^	16.49	16.65	16.87	17.03	16.63
Crude Ash	9.71	8.57	7.05	5.92	6.6

^a^ Metabolizable energy was a calculated value, while the others were measured values.

### 2.3. Collection of samples and recording of data

After a 14-day adaptation period, a 56-day feeding experiment began. The feed’s raw materials and leftovers from each group of experimental goats were collected and mixed evenly every 7 days. The collected feed samples were baked at 55°C to constant weight. The dried samples were crushed with a 1 mm sieve and stored at −20°C. The collected feed and leftover samples were used to determine the chemical composition. The digestion test was carried out by total fecal collection method on the 51st, 52nd, and 53rd days of the experimental period. About 500 g (5%) of fresh intestinal samples were collected at 7 o’clock every morning from each goat. After mixing the samples for 3 consecutive days, the samples were divided into two parts on average. One sample was mixed with 10% hydrochloric acid (10 mL) for every 100 g and evenly treated with nitrogen fixation. The other two intestinal samples were baked in an oven at 55°C to a constant weight, and the dried samples were crushed with a 1 mm sieve, marked, sealed, and stored at –20°C, and used to determine the digestibility of nutrients. On the 56th day of the experimental period, 2 h post morning feeding, 50 mL of rumen fluid samples were collected using an oral tube. In order to reduce saliva dilution of rumen fluid, the first sample was discarded before each sampling, and the filtrate was collected after four layers of gauze filtration. The contents of each goat cecum were collected after slaughter using pre-sterilized 5 mL lyophilized tubes and stored in liquid nitrogen, then transferred to the laboratory and stored in a −80°C refrigerator for gut microbiological analysis.

### 2.4. Index determination

#### 2.4.1. Determination of growth performance

On the 0th and 56th day of the experimental period, the experimental goats were weighed and recorded before morning feeding, and the average daily gain (ADG) of the experimental goats was calculated. Since the beginning of the formal trial, the daily feed intake and leftover feed of each experimental group were recorded in detail, and the total feed intake together with the feed efficiency were calculated according to the following formula:


Average daily gain g=Initial weight of experimental goat/Test days

Total feed intake of experimental days kg=

$$\begin{array}{l} \mathrm{Total feed volume during the test period}\\ -\mathrm{total feed leftover during the test period} \end{array}$$

$$\begin{array}{l} \mathrm{Total feed efficiency of the experimental period}\left(\%\right)\\ =\mathrm{total feed intake of experimental goats}/ \end{array}$$

$$\left(\begin{array}{l} \mathrm{final weight of the experimental goats}\\ -\mathrm{initial weight of experimental goats} \end{array}\right)$$

#### 2.4.2. Determination of feed composition and apparent digestibility

According to the procedure of the official Association of Analytical Chemists (Aboshora, 1990), the contents of dry matter (DM), crude protein (CP), and crude fat (EE) in feed raw material, leftover and hindgut samples were determined by wet chemical analysis in the Ruminant Nutrition Laboratory of Northeast Agricultural University. The contents of neutral detergent fiber (NDF) and acid detergent fiber (ADF) were analyzed according to the method of Van Soest et al. (1991). The results of NDF and ADF were expressed based on dry matter (DM). The apparent digestibility of nutrients was determined according to the hydrochloric acid insoluble ash (AIA) in feed and hindgut samples and calculated according to the method of Thonney et al. (1979).

#### 2.4.3. Determination of slaughter performance and mutton quality

On the last day of the trial period, four goats were randomly selected from each treatment group. Sixteen were transferred to the slaughter plant for the samples collected and data recorded. Each goat’s live weight and carcass weight before slaughter were recorded separately and used to determine the dressing percentage. The pH meter (pH meter: HI9125; Hanna Instruments, Pa-136 dova, Italy) electrode was inserted in the incision along the direction of the muscle fiber, and the data was recorded after the reading was stabilized. In addition, samples of the longest dorsal muscle were collected from the part of the goat between the 12th and 13th ribs of the left half of the carcass without fat for determining drip loss, total antioxidant capacity (T-AOC) and malondialdehyde (MDA). T-AOC and MDA were determined using commercial kits (A015-1-3 and A003-2; Nanjing Jiancheng Institute of Bio-engineering, 155 Nanjing). The calculation formula for dressing percentage and drip loss is as follows:

Dressing percentage % = Hot carcass weight/Live weight × 100%.

24 h: Drip loss % = (W_1_-W_2_) /W_1_× 100%.

48 h: Drip loss % = (W_1_-W_3_) /W_1_× 100%.

Each goat was divided into three square-sized samples of 3 × 3 × 3 cm and weighed as W_1_. The meat samples were suspended in sealing bags with fishing lines, stored in a refrigerator at 4°C, and weighed again after 24 h as W_2_ and 48 h as W_3_.

#### 2.4.4. Rumen microbiome and hindgut microbiome

Rumen and hindgut samples were sequenced by 16 s rDNA in Lianchuan Biotechnology Co., Ltd. A hindgut DNA kit (D4015, Omega, Inc., United States) was used, and nonnuclear water was treated as blank. Total DNA was eluted with 50 μL elution buffer and stored at −80°C until the sequencing was performed by LC-Bio Technology Co., Ltd. Polymerase chain reaction (PCR) analysis was performed in Zhejiang, China. Design of universal primer sequences based on the V3-V4 re-162 regions of the 16S rDNA amplified fragment: 341F (5′-CCTACGGGNGGCWGCAG-3′), 163805R (5′-GACTACHVGGGTATCTAATCC-3′). The 5′ end of the primer was marked by a special barcode and general sequencing primers. The template DNA 25 ng, PCR Premix 12.5 μL, primers 2.5 μL, and PCR grade water were adjusted to the final volume of 25 μL reaction solution before being amplified by PCR. The PCR conditions for amplification of prokaryotic 16S fragments are as follows: initial denaturation at 98°C for 30 s, denaturation at 98°C for 10 s, annealing at 54°C for 30 s, extension at 72°C for 45 s, and final extension at 72°C for 10 s. The PCR product was confirmed by 2% agarose gel electrophoresis. In the DNA extraction process, ultra-pure water was used as a negative control instead of a sample solution to rule out the possibility of false positive PCR results. PCR products were purified by AMPure XT beads (Beckman Coulter Genomics, Danvers, MA, United States) and quantified by Qubit (Invitrogen, United States). The size and quantity of the amplified sublibrary were evaluated by Agilent 2100 biological analyzer (Agilent Technologies, United States) and Illumina sequencing platform KAPA library quantitative kit (KAPA Biosciences, Woburn, MA, United States). These libraries are sequenced on the NovaSeq PE250 platform.

### 2.5. Statistical analysis

Growth parameters data were analyzed by one-way analysis of variance (ANOVA) and the Duncan range test. The data of the Alpha diversity index were expressed as mean ± standard deviation, and ANOVA analysis of the Fisher minimum significant difference (LSD) test was used. The statistical analysis was performed using the SPSS version 25.0 of Windows (SPSS Inc., Chicago, IL, United States). *p* < 0.05 is statistically significant, and 0.05 < *p* < 0.10 is a significant trend.

Samples were sequenced on the Illumina NovaSeq platform according to the manufacturer’s recommendations (LC-Bio Technologies). Paired-end reads were performed on the sample based on its unique barcode and truncated using the barcode and primer sequences. Peer reads were merged using FLASH. Quality filtered raw reads according to fqtrim (v0.94) under specific filter conditions to obtain high-quality clean labels. Chimeric sequences were screened using VSEARCH software (v2.3.4). The feature table and feature sequence were obtained after deduplication using DATA2. Beta diversity was normalized to the same sequencing depth by randomly removing aligned reads. The feature abundance then normalized the relative abundance of each sample according to the SILVA (release 132) classifier. Beta diversity was calculated by QIIME2 and plotted by the R package. The correlation heatmap was analyzed by plotting the Spearman correlation matrix using the R package (v3.6.3). Sequence alignment was performed using Blast, and the SILVA database was used to annotate the characteristic sequences for each representative sequence. Other graphs were generated using the R package (v3.5.2).

## 3. Results

Table 2 shows the growth performance of different roughage proportions, and variance analysis was carried out with the initial body weight as a covariate. The results showed that feeding whole alfalfa could significantly reduce the daily weight gain of fattening goats, which was the most serious, up to 19.21% compared with the OA73 group. Although there was no significant change in feed intake, the feed conversion rate of the whole alfalfa group decreased significantly by 30.24–36.47% compared with the other groups. Table 3 shows the results of nutrient metabolism in this study. In general, with the increase in alfalfa content, the digestibility of other nutrients increased significantly, except for fat digestibility. Compared with whole oats, the CP digestibility of whole alfalfa increased by 8.25%, while the DM, NDF, and ANF increased by 14.9, 23.4, and 37.2%, respectively (*p* < 0.05).

**Table 2:** Effects of oat and alfalfa on the growth of fattening goats.

	Treatments^1^				SME	*P*-value
Item	Oat	OA73	OA37	Alfalfa
Initial weight, kg	21.80	22.26	21.88	22.40	0.33	0.909
Final weight, kg	26.16^a^	26.68^a^	26.30^a^	25.98^b^	0.29	<0.001
Average daily gain, g	77.90^a^	78.80^a^	79.02^a^	63.84^b^	3.51	0.026
Daily feed intake, kg	1.01	0.95	1.03	1.09	0.02	0.336
Feed conversion ratio, kg/kg	14.02^a^	13.38^a^	13.45^a^	18.26^b^	0.90	0.033

^1^Treatments: Oat, roughage composition = 100% oats; OA73, roughage composition = 70% oats & 30% alfalfas; OA37, roughage composition = 30% oats & 70% alfalfas; Alfalfa, roughage composition = 100% alfalfas. ^a,b^ With row, different superscripts indicate differences between treatments (*P* < 0.05).

**Table 3:** Effects of oat and alfalfa on apparent digestibility of fattening goats (%).

	Treatments^1^				SME	*P-*value
Item	Oat	OA73	OA37	Alfalfa
Dry matter	77.74^a^	76.50^a^	84.44^ab^	89.34^b^	1.70	0.012
Crude protein	86.82^a^	85.47^a^	91.11^ab^	93.98^b^	1.11	0.020
Ether extract	86.73	84.70	91.56	92.83	1.29	0.128
Neutral detergent fiber	70.49^a^	72.13^a^	80.44^ab^	86.98^b^	2.17	0.010
Acid detergent fiber	64.21^a^	67.72^ab^	79.74^bc^	88.08^c^	2.82	0.001

^1^Treatments: Oat, roughage composition = 100% oats; OA73, roughage composition = 70% oats & 30% alfalfas; OA37, roughage composition = 30% oats & 70% alfalfas; Alfalfa, roughage composition = 100% alfalfas. ^abc^ With row, different superscripts indicate differences between treatments (*P* < 0.05).

Table 4 shows the meat production traits of fattening goats fed with different proportions of roughage. The live weight was considered a covariable in the slaughtering and meat quality analysis. The results showed that the dressing percentage, pH, and drip loss of fattening goats had no significant difference (*p* > 0.05). Interestingly, T-AOC showed a trend with increased alfalfa content (0.05 < *p* < 0.10), and MDA was significantly lower than the other treatments (*p* < 0.05). The antioxidant activity of fattening goat meat fed with the high alfalfa group was significantly higher than that of the low alfalfa group.

**Table 4:** Effects of oat and alfalfa on carcass traits of fattening goats and quality of mutton.

	Treatments^2^				SME	*P*-value
Item^1^	Oat	OA73	OA37	Alfalfa
Carcass weight, kg	14.25	12.37	13.90	14.07	0.27	0.106
Dressing percentage, %	52.43	49.22	52.17	54.81	0.01	0.672
pH	6.62	6.68	6.58	6.78	0.06	0.784
Drip loss-24 h, %	0.73	0.59	0.73	0.50	0.06	0.631
Drip loss-48 h, %	0.86	0.75	0.93	0.59	0.06	0.239
T-AOC, U/mL	1.33^a^	1.89^a^	4.08^b^	3.25^b^	0.14	0.050
MDA, nmol/mL	4.20^a^	3.74^ab^	3.64^ab^	3.37^b^	0.07	0.035

^1^Item: T-AOC, total antioxidant capacity; MDA, malondialdehyde. ^2^Treatments: Oat, roughage composition = 100% oats; OA73, roughage composition = 70% oats & 30% alfalfas; OA37, roughage composition = 30% oats & 70% alfalfas; Alfalfa, roughage composition = 100% alfalfas. ^ab^ With row, different superscripts indicate differences between treatments (*p* < 0.05).

**Figure 1:**
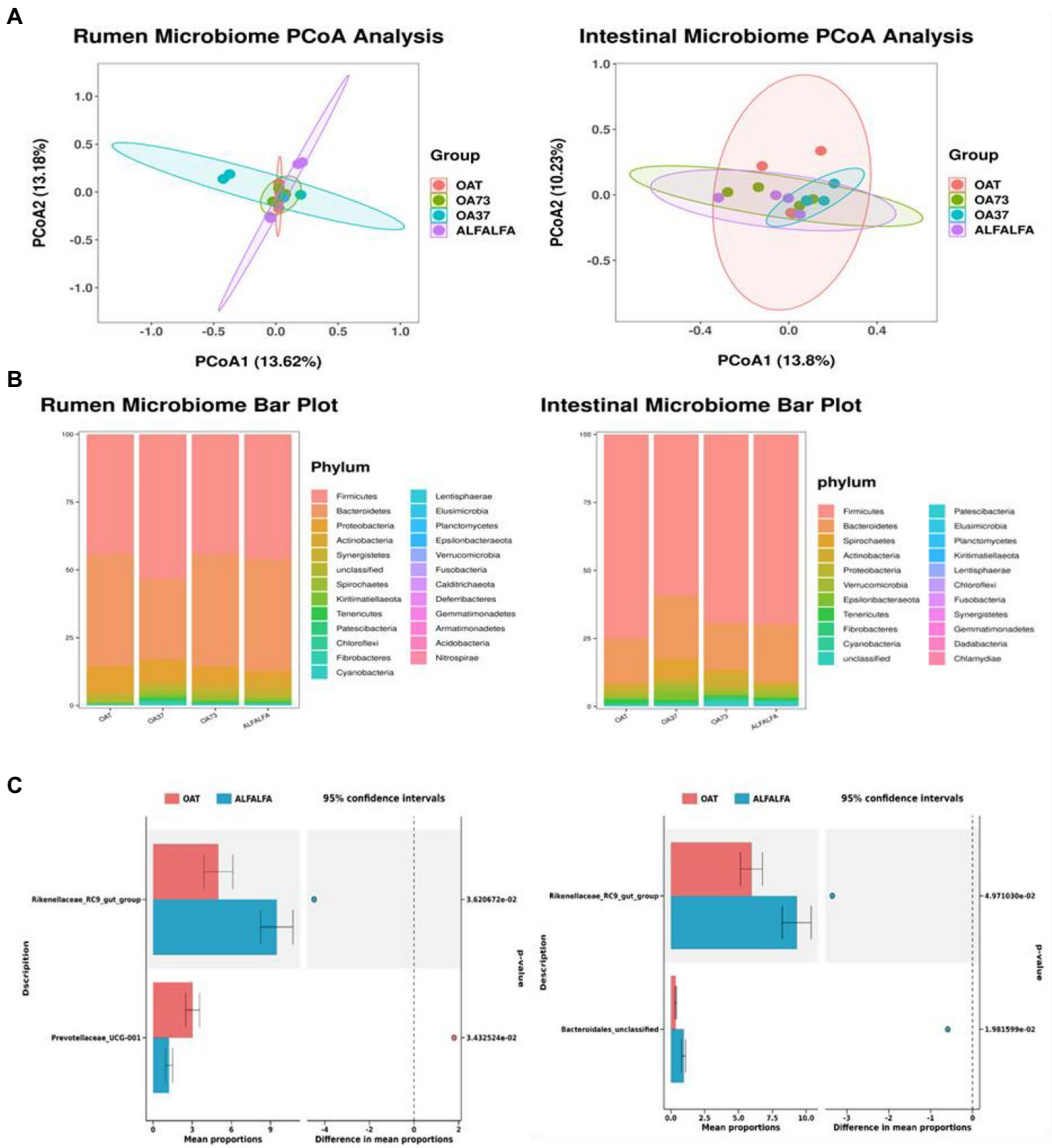
Classification of microbiome composition in posterior gut and rumen. **(A)** Principal component analysis. **(B)** Differences in microbiome phylum levels among groups. **(C)** Differences in microbiome genus levels between whole oat and whole alfalfa groups.

To investigate the effect of alfalfa and oat hay diet mix on beta diversity, we used unweighted UniFrac distances to characterize bacterial communities across all rumen samples (Figure 1). PCoA map showed that the bacterial community composition of these groups was not significantly separated from each other. A total of 25 phyla and 362 genera were found in the rumen, and 22 phyla and 369 genera in the posterior intestine. At the rumen phyla level, seven phyla were referred to as detection phyla (relative abundance >1% in at least one group). The most abundant phyla were Firmicutes, followed by Bacteroidetes and Proteobacteria. The total abundance of these three phyla exceeded 90%, but there was no significant difference among all groups (*p* > 0.05). At the genus level, 26 genera were considered detectable (relative abundance >1% in at least one group). Classified mainly as *Prevotella_1*, *Muribaculaceae_unclassified*, *Ruminococcus_2*, *Succiniclasticum, VeillonellACEAE_UCG-001*, *Rikenellaceae_RC9_gut_group*, *Succinivibrio, Ruminococcaceae_NK4A214_group*, *Christensenellaceae_R-7 _group*, *Prevotella*, *Prevotellaceae_UCG-001*, *F082_unclassified, Lachnospiraceae_unclassified*, the total abundance of these 13 genera exceeded 60%.

**Figure 2:**
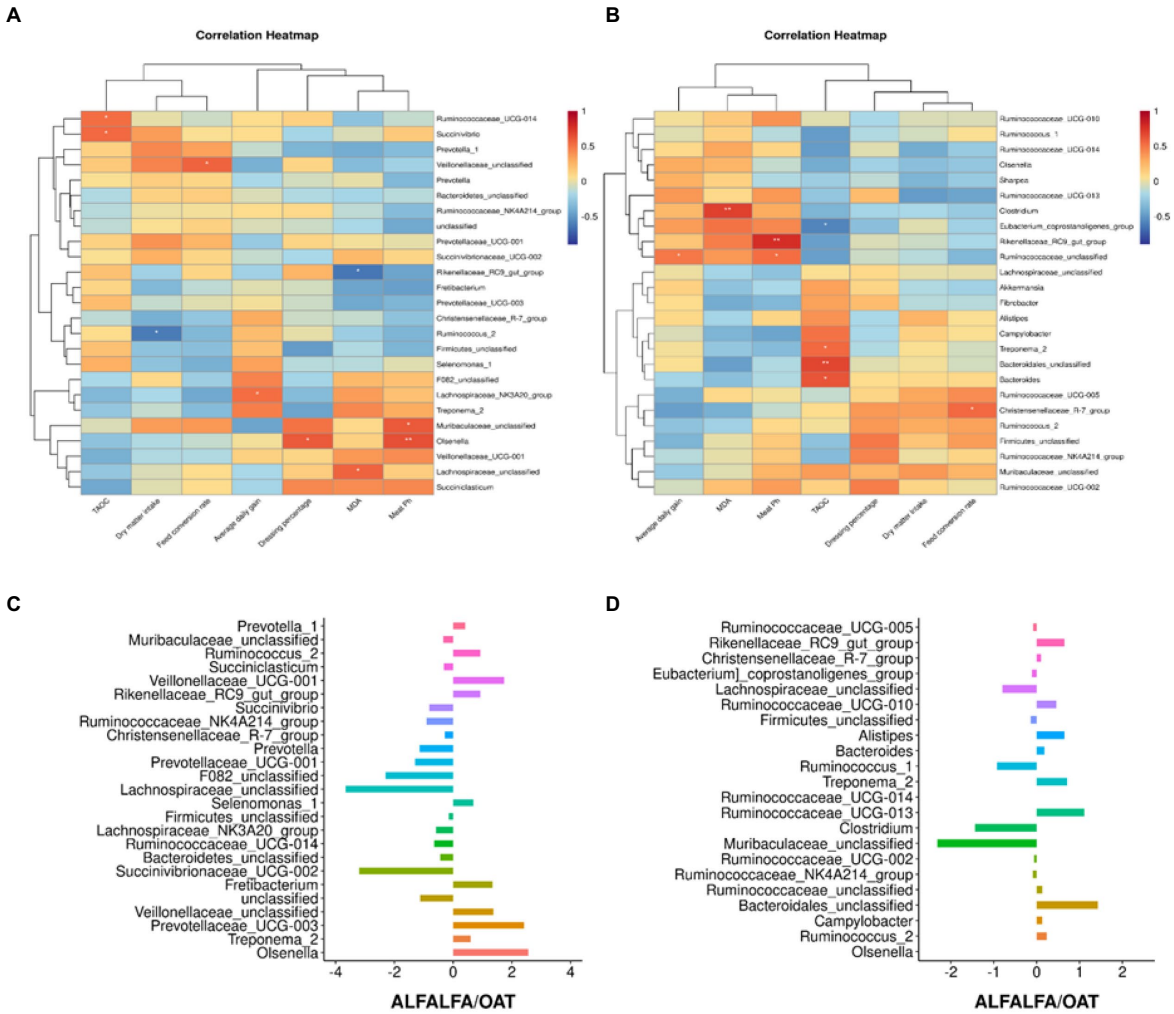
Effects of changes in the dominant microbiome of alfalfa and oat on growth performance and meat quality. **(A)** Heatmaps of Pearson’s correlations between rumen dominant genera and growth performance and meat quality. **(B)** Heatmaps of Pearson’s correlations between intestinal dominant genera and growth performance and meat quality. **(C)** Differential rumen genus microbiome between alfalfa and oat. **(D)** Differential intestinal genus microbiome between alfalfa and oat, *p < 0.05, **p < 0.01.

In the results of genus level significance, this study compared the differences of the microbiota at the genus level between the alfalfa and the oat groups, and the results showed that the *Rikenellaceae_RC9_gut_group* in the rumen and posterior gut was significantly higher than that in the oat group. *Bacteroidales_unclassified* in the back intestine of the alfalfa group was significantly higher than that of the oat group, but there was no significant change in the level of other rumen microbiota genera. Pearson correlation analysis was performed to investigate further the correlation between dominant microbial genera and growth performance and meat quality (Figure 2). This study focused on the correlation between microbiota, T-AOC, and MDA of meat. In the rumen, R*uminococcaceae_UCG-14* and *Succinivbrio* are significantly positively correlated. *Olsenella* was positively correlated with the pH of meat. *Lachnospiraceae_unclassified* was positively correlated with MDA, while *Rikenellaceae_RC9_gut_group* was negatively correlated with MDA. In addition, *Olsenella* was significantly correlated with meat pH and dressing percentage, while other genera had no significant differences in growth performance, meat quality, and fatty acid profile (*p* > 0.05).

In the back intestinal flora, the results of this study showed that *Clostridium* was positively correlated with MDA. Meanwhile, T-AOC could be influenced by *Bacteroidales_unclassified*, Treponema_2, and Bacteroides, which have a negative relationship with *Eubacterium_coprostanoligenes_group*. Meat pH was significantly positively correlated with *Rikenellaceae_RC9_gut_group* and *Ruminococaceae_unclassified* in the hindgut tract. In order to further explore the differences in growth performance and meat quality of mixed hay, the gut microbiota of the alfalfa and star wheat groups were compared. *Muribaculaceae_unclassified* and *Clostridium* of the oat group were twice as high as those of the Alfalfa. In contrast, *Campylobacter* was less than 1 time that of the alfalfa group.

## 4. Discussion

This study showed the effects of the different mixed ratios of oat and alfalfa on fattening goats’ growth and slaughtering performance. The results showed that a high proportion of alfalfa in roughage could improve the antioxidant activity of mutton, but whole alfalfa could reduce daily gain and feed conversion rate. Like another study, the total use of alfalfa in the diet did not significantly improve the daily gain of livestock (Tang et al., 2021). In addition, this might be related to the high content of ADF in alfalfa. The high level of ADF tends to increase the ruminal activity and its fermentation retention time in the rumen, increasing its thermal power consumption. And it was associated with the production of large amounts of gas by rumen microbial fermentation, such as the increased relative abundance of *Prevotella* in this study. However, the Pordomingo study reported that alfalfa could improve the quality of beef, especially the cooking loss and sensory evaluation indexes of meat, including juiciness, tenderness, and beef flavor (Pordomingo et al., 2022). Significantly, in this study, supplementing oats based on alfalfa could increase the daily weight gain of mutton sheep.

Live weight is a crucial economic character in animal husbandry, which is the most important factor affecting the market price in China (Zhou et al., 2012). Therefore, daily gain and feed conversion rate directly determine the farmers’ income. In this study, the daily gain and feed conversion rate of the whole alfalfa group were significantly lower than those of other groups, which indicated that alfalfa hay alone could not be effectively converted into weight gain, but mixing it with an oat diet could further improve the digestive function of animals. Castells’s experiment found that the average daily gain of calves fed with oats was 63% higher than that of alfalfa diets (Castells et al., 2013). Interestingly, our results are similar to those of Doran (Doran et al., 2007). The digestibility of alfalfa is significantly higher than that of oats. Oats are thought to contain more non-structural carbohydrates (Sánchez-Velázquez et al., 2021), have more pectin than alfalfa, and can be quickly degraded and absorbed into energy in the rumen (Evan et al., 1990), but their growth performance is also affected by climate, environment, feeding methods, and other factors (Pereira et al., 2018; Cremilleux et al., 2022). More dry matter is converted into feces, urine, and other forms of energy loss.

The core microflora of the phylum is Bacteroidetes, Firmicutes, and Proteobacteria, which account for about 90% of the bacterial species (Anderson et al., 2017; Guo et al., 2020). Bacteroidetes and Firmicutes are the most abundant and active flora members involved in carbohydrate and protein degradation (Xue et al., 2021). The main function of Bacteroides is to degrade many kinds of plant polysaccharides, improve the efficiency of nutrient utilization, and enhance the host’s immunity (Delgado et al., 2019). Phaeophyta plays another essential role in the degradation of fiber and cellulose, related to the decomposition of polysaccharides and energy utilization (Delgado et al., 2019). There was no significant difference in rumen hilum level among the groups in this study. The change of roughage did not change the bacterial richness and taxonomic composition but had a different relative abundance at the genus level, which was consistent with other reports (Schären et al., 2017; Liang et al., 2021) and may reflect the particular niche related to dietary fiber digestion.

Many studies have reported that increasing the proportion of alfalfa in the diet can improve the meat quality traits of animals. This study confirmed that the increase of alfalfa content significantly increased the T-AOC of goat meat and decreased the MDA content, which was the same as that of Su et al. (2022). The malondialdehyde content of alfalfa powder in the Tibetan diet decreased by 16.5%. Plant active components, including flavonoids, saponins, and polysaccharides, contain antioxidant-free, radically active groups which can scavenge free radicals by supplying hydrogen to react with free radicals (Prommachart et al., 2021). This reduces the raw materials for synthesizing free radicals and increases the enzymes that decompose it to play an antioxidant role. Flavonoids, saponins, and polysaccharides all increased T-AOC and decreased MDA content in different experimental animals such as cows and mice, which means that the combined action of plant active components in alfalfa may be an important reason for improving the ability of the antioxidant defense system (Zhan et al., 2017; Xie et al., 2019).

This study discussed the correlation analysis between hindgut and rumen microorganisms with growth and slaughtering performance. The results showed that the microorganisms in the body could affect the antioxidant capacity of goat muscle. In this study, *Bacteroidales_unclassified* and *Bacteroidales* were significantly or very significantly positively correlated with T-AOC, and *Bacteroidales_unclassified* in the alfalfa group was significantly higher than in the oat group, suggesting that B*acteroidales_unclassified* may be the key to improving meat antioxidant activity in alfalfa diet. It is well known that *Bacteroidale* plays a key role in rumen carbon transformation. Although *Bacteroidales_unclassified* lacks a clear and complete genome, its biological function may have the same ability to release hemicellulose monosaccharides as most other members of *Bacteroides*. Through genome metabolic analysis, *Solden* revealed various ways to ferment hemicellulose monosaccharides into short-chain fatty acids (SCFA), especially the increase in butyrate content (Solden et al., 2017). A recent study has shown that butyric acid can increase the T-AOC of the body by increasing the activity of SOD and CAT, clearing the levels of free radicals and MDA in the host (Li et al., 2022). This is an important way to improve the body’s antioxidant activity in the hindgut environment.

There was a significant correlation between *Clostridium* and MDA in the hindgut. In this study, the *Clostridium* in the hindgut of the alfalfa group was significantly lower than that of the oat group. Similarly, Wu added – to the diet of yak with alfalfa hay significantly decreased the content of *Clostridium XVIII* and LPS, decreased the inflammatory reaction, and improved the liver health of yaks (Wu et al., 2019). The increase of *Clostridium* in the intestinal tract can cause Clostridial abomasitis and enteritis, mainly characterized by the necrosis of Bacillus or intestinal mucosa caused by the exotoxin produced by *Clostridium* perfringens or *Clostridium* difficulty in the gastrointestinal lumen. The proliferation of *Clostridium* perfringens in the gastrointestinal tract of ruminants is related to the increase in carbohydrates or proteins and the changes in gastrointestinal motility (Simpson et al., 2018). In this study, a high-concentrate diet may increase the abundance of *Clostridium*, while the addition of alfalfa hay decreases the abundance of *Clostridium*, which is responsible for the decrease of MDA. In addition, the results showed that the pH of goat meat was significantly correlated with *Olsenella* in the rumen and *Rikenellaceae_RC9_gut_group* in the intestine, which meant that different proportions of alfalfa and oat changed the microflora and affected the meat quality.

## 5. Conclusion

A high proportion of alfalfa in roughage can improve the antioxidation of mutton and the digestibility of nutrients, but whole alfalfa can reduce daily weight gain and feed conversion rate. In addition, there is no significant effect of different proportions of forage on carcass traits. Feeding alfalfa could increase the abundance of *Bacteroidales_unclassified* and decrease the abundance of *Clostridium*. These microorganisms were strongly correlated with the antioxidant activity of mutton. The different roughage composition affects the intestinal microflora, thus improving livestock performance as edible products.

## Data availability statement

The data presented in the study are deposited in the NCBI Sequence Read Archive (SRA), accession numbers PRJNA880490 and PRJNA880492.

## Ethics statement

The animal study was reviewed and approved by the Northeast Agricultural University Animal Care and Use Committee (Harbin, China) (protocol code NEAU- [2011]-9) and were in accordance with the recommendations of the academy’s guidelines for animal research. Written informed consent was obtained from the owners for the participation of their animals in this study.

## Author contributions

YS: methodology, visualization, investigation, formal analysis, and writing – original draft. TH: investigation. QY: visualization. CZ: project administration. YZ: supervision and writing – review and editing. YS: methodology and writing – review and editing. LX: funding acquisition, methodology, and writing review and editing. All authors contributed to the article and approved the submitted version.

## Funding

This study was supported by the National Key Research and Development Program of China (2021YFD1300504), and this research was funded by Research and Demonstration of Water-saving and Efficient Production and Utilization Technology of High-quality Forage Material, grant number KJXM-EEDS 2020010-04 and China Agriculture Research System of MOF and MARA (CARS36).

## Conflict of interest

The authors declare that the research was conducted in the absence of any commercial or financial relationships that could be construed as a potential conflict of interest.

## Publisher’s note

All claims expressed in this article are solely those of the authors and do not necessarily represent those of their affiliated organizations, or those of the publisher, the editors and the reviewers. Any product that may be evaluated in this article, or claim that may be made by its manufacturer, is not guaranteed or endorsed by the publisher.
